# Comparative assessment of health-related quality of life among hypertensive patients attending state and federal government teaching hospitals in Ekiti State, Nigeria

**DOI:** 10.1016/j.dialog.2022.100069

**Published:** 2022-11-02

**Authors:** Tope Michael Ipinnimo, Kayode Rasaq Adewoye, Kabir Adekunle Durowade, Olusegun Elijah Elegbede, John Olujide Ojo, Bolade Folasade Dele-Ojo, Olarinde Jeffrey Oluwademilade, Oladele Ademola Atoyebi, Taofeek Adedayo Sanni, Olumide Temitope Asake, Blessing Waibi Daramola, Adetunji Olamide Fadipe

**Affiliations:** a Department of Community Medicine, Federal Teaching Hospital, Ido-Ekiti, Nigeria; b Department of Community Medicine, Afe Babalola University, Ado Ekiti, Nigeria; c Department of Medicine, Ekiti State University Teaching Hospital, Ado-Ekiti, Nigeria; d Department of Medicine, Federal Teaching Hospital, Ido-Ekiti, Nigeria; e Department of Medicine, Afe Babalola University, Ado-Ekiti, Nigeria; f University of British Columbia, Canada

**Keywords:** Health-Related Quality of Life, Hospitals, Hypertension, Nigeria

## Abstract

**Background:**

Hypertension is a serious health problem and it is one of the diseases that impair health-related quality of life. The central tenet of care should be to improve health-related quality of life and overall well-being and not just be limited to improving clinical outcomes. This study assesses and compares health-related quality of life and its predictors among hypertensive patients in two government hospitals in Ekiti State, Nigeria.

**Methods:**

This was a comparative cross-sectional study involving 440 hypertensive patients (220 in each group), recruited using a systematic sampling technique within the hospitals. Data on socio-demographic, economic and clinical characteristics including the cost of care for hypertension were collected from the patients. The WHOQoL-BREF questionnaire was used to assess health-related quality of life. Data were entered and analyzed using IBM SPSS Statistics for Windows, Version 22.0.

**Results:**

All domains of health-related quality of life were better among patients in federal government teaching hospitals, however, only the physical (*T* = −7.932, *p* < 0.001) and overall (*T* = −2.783, *p* = 0.006) domains were of statistical significance. An inverse relationship between cost and health-related quality of life was found in the two hospitals (State: *r* = −0.224, *p* = 0.001; Federal: *r* = −0.378, *p* < 0.001). Identified predictors of health-related quality of life were age, locality of residence, income, number of complications, exercise and smoking in both hospitals. Other predictors were marital status, living arrangement, occupation, number of medications, and involvement in religious and spiritual activities among patients in the state government teaching hospital; household size, length of diagnosis, and indirect cost among patients in the federal government teaching hospital.

**Conclusion:**

There is a need to support hypertensive patients in the state government teaching hospitals to reduce the inequality of low health-related quality of life among them. Identified predictors should be taken into consideration when putting in place policies that will improve the health-related quality of life of these patients.

## 1 Introduction

The concept of health-related quality of life (HRQoL) was first mentioned in 1980 and has been introduced into literature since then [1,2]. Defining HRQoL has been challenging, and the concept and its determinants have evolved over the past decades [[1], [2], [3]]. It is described as a person's perceived quality of life representing satisfaction in areas of life likely to be affected by health status [3]. In addition, the Centre for Disease Control defined HRQoL based on the synthesis of literature as a person's or group's perceived physical and mental health over time [1].

Hypertension is one of the diseases that may impair the HRQoL of patients [[4], [5], [6]]. Apart from the physical and physiological impact of the disease, the psychological stress following the awareness of being ill often affects the quality of life and may lead to decreased satisfaction with daily living [3,7]. The socio-economic burden of hypertension, its comorbidities and the fear of developing life-threatening complications may all have a negative effect on patient's day-to-day life [3]. The goal of treatment should be to improve patients' quality of life and overall well-being and not just be limited to improving their clinical outcomes.

Studies in Brazil [4,8] and Croatia [9] have shown that hypertensive patients have a lower HRQoL than the general population. Similar findings were also seen in patients with other chronic diseases [8]. Studies from Palestine, Nepal, China and other parts of Asia revealed similar results as well [[10], [11], [12], [13], [14]]. In Africa, literatures have shown that hypertension has a significant effect on HRQoL [5,15]. Studies from a federal government teaching hospital (FTH) in Edo State, Nigeria and another from two tertiary hospitals in Bayelsa State, Nigeria, revealed slightly lower HRQoL scores among hypertensive patients than the general population [16,17].

In a FTH in Oyo State, Nigeria, a mean total HRQoL score of 63.59 ± 8.91 was recorded among hypertensive patients. Drug use was found to worsen the HRQoL of these patients, but blood pressure control had no effect [5]. There have been inconsistent reports on how the number of medications affects the HRQoL of hypertensive patients. Some studies revealed that drug use or increasing numbers of drugs worsen the HRQoL [5,10]; while some others reported a better HRQoL with an increasing number of antihypertensive medications [12]. Another study in Brazil revealed no association between the number of antihypertensive drugs, their doses and HRQoL of hypertensive patients [4].

Furthermore, studies have found that age [3,11], gender [3,12] and blood pressure control [4,5] do not affect the HRQoL of hypertensive patients. However, several studies have shown that male gender [4,5,[8], [9], [10], [11],^13^], younger age [4,8,10,12,13], higher income and socio-economic status [4,5,[8], [9], [10], [11], [12],^16^], higher level of education [[3], [4], [5],[8], [9], [10], [11], [12],^16^], being employed [3,[8], [9], [10],^13^], being married or living with a spouse [4,10,12,13], monogamy and small family size [5,12], living in urban areas [3,8,13] have positive effect on the HRQoL of hypertensive patients. Other positive predictors found in previous studies were compliance with low salt diet advice [12], engaging in physical activities [13], cessation of smoking and alcohol [13], controlled blood pressure [16], shorter duration of disease from the time of diagnosis [4,10], low symptom count and absence of complication [5,9], normal BMI [10] and absence of co-morbidities [[9], [10], [11]]. Additionally, a high level of involvement in spiritual and religious activities was associated with a better HRQoL score [18].

Studies on the relationship between cost of care and HRQoL especially among hypertensive patients are limited. However, an inverse association was found between these two variables among stroke survivors, cancer survivors and patients with ankylosing spondylitis [[19], [20], [21]]. This study also intends to explore the relationship between HRQoL and cost of care in addition to other socio-demographic, economic and clinical factors. Evaluation of these relationships could enable decision-makers to understand the financial factors that influence HRQoL in hypertensive patients. Subsequently, this knowledge will enable them to recognize aspects of the healthcare cost of hypertension treatment that need intervention to improve treatment outcomes.

Most of the hospital morbidity and mortality in Ekiti State were due to hypertension and its complications [22] and to the best of our knowledge, no study in Ekiti State has assessed the HRQoL of this disease, especially one comparing the State Government Teaching Hospital (STH) and the FTH. Thus, it is important to compare HRQoL among hypertensive patients in the STH and the FTH since a large proportion of hypertensive patients attend these hospitals [22,23]. Moreover, these hospitals differ in their infrastructure and equipment, the strength of their human resources as well as their budget. Assessment and comparison of HRQoL of hypertensive patients treated at STH and FTH could help detect the gaps in the care of patients patronizing these hospitals and can inform the implementation of appropriate interventions.

## 2 Materials and methods

### 2.1 Study design and setting

This was a comparative cross-sectional study, carried out among hypertensive patients in STH and FTH in Ekiti State, Nigeria. The STH is located in an urban area with about 1350 members of staff while the FTH is located in a semi-urban area with over 3000 staff strength. The STH depends on the monthly subvention from Ekiti State Government, which is often much less than the annual budgetary allocation given by the Federal Government to FTH [24,25]. This study was conducted between October and December 2019.

### 2.2 Participants and methods of selection of participants

Study participants included all adult hypertensive patients who had been on treatment for at least 3 months while those who were too ill to respond on the day of clinic were excluded. The minimum sample size for this study was determined for each group (STH and FTH) using the formula for calculating sample size comparing two proportions [26]. A 95% confidence interval, 80% power, 24%-prevalence of hypertension in a FTH in Nigeria [27], 13%-prevalence of hypertension in a STH in Nigeria [22] and 10% adjustment for non-response gave a sample size of 220, thus making 440 hypertensive patients (220 each in STH and FTH) that were approached and interviewed.

Hypertensive patients from the hospitals were selected using a systematic sampling technique based on their arrival at the clinics. The first patients (among the first four in STH and the first two in FTH) were selected by ballot; subsequently, every fourth patient was selected in the STH while every second patient was selected in the FTH based on the calculated sampling interval. This sampling interval (4 for STH and 2 for FTH) was obtained by dividing average clinic attendance in 10 clinic days in each hospital by 220. These average clinic attendances were estimated from past clinic records. Selected consented patients were interviewed after their clinic consultations.

### 2.3 Variables and measurements

A semi-structured, interviewer-administered questionnaire was used to collect data on socio-demographic, economic and clinical characteristics including cost of care of hypertension. Four research assistants (medical students in the clinical stage of their training) were employed and trained on how to appropriately administer the questionnaire and to ensure reliability and prevent bias. Clinical information collected included duration of diagnosis, number of medications, complications and co-morbidities. The weights of patients were measured using a well-calibrated scale (Hana bathroom scale model BR) with the patients barefooted and weight evenly distributed while height was measured using a portable stadiometer with the patients standing erect barefooted against a wall and measurement taken from the heels to the vertex with the head in the Frankfurt horizontal plane [28]. The weight and height of patients were measured to the nearest 0.1 kg and 0.1 m respectively and were used to estimate their body mass index (BMI) [29]. Additionally, patients were asked about their blood pressure measurement during the current clinic visit and this was verified from their medical records. History of alcohol intake, smoking, compliance with low salt diet, exercise, as well as intake of fruits and vegetables were also taken. The socioeconomic status was measured by dividing the patients into quintiles based on their consumption expenditure. This gave 5 groups consisting of 20% of the population each through poorest, poor, average, rich and richest.

The WHOQoL-BREF questionnaire [30] was used to assess the HRQoL of patients. The WHOQoL-BREF does not have floor and ceiling effects [31] and its psychometric properties supported its use for hypertension [5]. It contains a total of 26 questions with 24 questions assessing four main domains of HRQoL namely; physical health, psychological, social relationship and environmental status. Also, two items examined separately the overall domain (global value). Each question was scored on a scale of 1–5 (scores were scaled in a positive direction except for 3 negatively phrased questions which were reversed) and then transformed to 0–100 scale, with 0 being the worst possible health status, and 100 the best. The score of items within each domain was used to calculate the domain score. If an individual had less than 80% of data present from an assessment, the assessment was discarded.

### 2.4 Data management and statistical analysis

Data were entered and analyzed using the IBM SPSS Statistics for Windows, Version 22.0 (IBM Corp., Armonk, N.Y., USA). Socio-demographic, economic, clinical and other categorical variables were presented using frequency tables and percentages. Mean and standard deviation were used to summarize age while median and interquartile range were used for patient's monthly income and cost of care. For HRQoL, mean scores and standard deviation in all domains were calculated. Mean HRQoL scores of patients were compared using the independent Student-*t*-test while the distribution of qualitative variables between groups was compared using chi-square test. Association between HRQoL and other continuous variables was determined using Pearson correlation coefficient. Multiple linear regression was used to determine the predictors of HRQoL as the dependent variable and socio-demographic, economic, clinical and other characteristics as the independent variable in STH and FTH. *P* < 0.05 was taken as statistical significance at both bivariate and multivariate levels.

### 2.5 Ethical consideration

Ethical approval for the study was sought and obtained from the Ethics and Research Review Committee of Federal Teaching Hospital, Ido-Ekiti, Nigeria. Written informed consent was obtained from all the research subjects. The procedures in this study adhered to the principle of Helsinki Declaration.

## 3 Results

The mean age and standard deviation of the STH patients was 58.4 ± 13.4 years while that of FTH patients was 57.8 ± 13.2 years (*T* = 0.442, *p* = 0.659). There was a statistically significant difference in the level of education (X^2^ [df:3] = 14.501, *p* = 0.002), household size (X^2^ [df:1] = 13.733, *p* < 0.001), locality of residence (X^2^ [df:2] = 58.616, *p* < 0.001), living arrangement (X^2^ [df:1] = 8.598, *p* = 0.003), occupation (X^2^ [df:2] = 14.272, *p* = 0.001), socioeconomic status (X^2^ [df:4] = 40.732, *p* < 0.001), number of complications (X^2^ [df:2] = 7.024, *p* = 0.030), number of days of exercise (X^2^ [df:1] = 21.359, *p* < 0.001), BMI (X^2^ [df:3] = 9.630, *p* = 0.022), the median direct cost (M = 14,419.5, *p* < 0.001) and median total cost of care of hypertension (M = 15,062.0, p < 0.001) among patients in STH and FTH. FTH had a significantly higher proportion of patients that were living with someone (STH, 168[82.4%]; FTH, 186[92.1%]), with no complication (STH, 99[48.5%]; FTH, 124[61.4%]), with normal BMI (STH, 57[27.9%]; FTH, 79[39.0%]) as well as had lower median direct cost (STH, ₦10,000 ± 17,313; FTH, ₦5550 ± 8316) and median total cost of care (STH, ₦13,200 ± 18,400; FTH, ₦7750 ± 8600) of hypertension. Details of socio-demographic and other characteristics are in Table 1.

**Table 1:** Socio-demographic, clinical and other characteristics of patients.

Variables	Health Facility			*X*^*2*^	*p*-value
	STH (%) *n* = 204	FTH (%) *n* = 202	Total (%) *N* = 406		
*Mean age±SD (years)*	*58.4±13.4*	*57.8±13.2*	*58.1±13.3*	0.442*	*0.659*
Sex					
Male	93 (45.6)	94 (46.5)	187 (46.1)	0.037	0.848
Female	111 (54.4)	108 (53.5)	219 (53.9)		
Level of Education					
No formal education	27 (13.2)	45 (22.3)	72 (17.7)	14.501	**0.002**
Primary education	36 (17.6)	40 (19.8)	76 (18.7)		
Secondary education	46 (22.5)	58 (28.7)	104 (25.7)		
Tertiary education	95 (46.6)	59 (29.2)	154 (37.9)		
Marital Status					
Unmarried	50 (24.5)	57 (28.2)	107 (26.4)	0.719	0.396
Married	154 (75.5)	145 (71.8)	299 (73.6)		
Family Type					
Monogamy	152 (74.5)	147 (72.8)	299 (73.6)	0.158	0.691
Polygamy	52 (25.5)	55 (27.2)	107 (26.4)		
Household Size					
1–4 persons	137 (67.2)	99 (49.0)	236 (58.1)	13.733	**<0.001**
>4 persons	67 (32.8)	103 (51.0)	170 (41.9)		
Locality of Residence					
Rural	40 (19.6)	90 (44.6)	130 (32.0)	58.616	**<0.001**
Semi-urban	26 (12.7)	52 (25.7)	78 (19.2)		
Urban	138 (67.7)	60 (29.7)	198 (48.8)		
Living Arrangement					
Living alone	36 (17.6)	16 (7.9)	52 (12.8)	8.598	**0.003**
Not living alone	168 (82.4)	186 (92.1)	354 (87.2)		
Occupation					
Formal	82 (40.2)	61 (30.2)	143 (35.3)	14.272	**0.001**
Informal	57 (27.9)	93 (46.0)	150 (36.9)		
Retired/Unemployed	65 (31.9)	48 (23.8)	113 (27.8)		
*Median income±IQR (₦)*	43,000 ± 83,725	41,400 ± 46,850	41,650 ± 61,750	18,914.00^M^	0.153
Socioeconomic Status					
Poorest	31 (15.2)	50 (24.8)	81 (19.9)	40.732	**<0.001**
Poor	41 (20.1)	41 (20.3)	82 (20.4)		
Average	24 (11.8)	57 (28.2)	81 (19.9)		
Rich	61 (29.9)	20 (9.9)	81 (19.9)		
Richest	47 (23.0)	34 (16.8)	81 (19.9)		
Length of Diagnosis					
1–4 years	111 (54.4)	101 (50.0)	212 (52.2)	0.792	0.374
>4 years	93 (45.6)	101 (50.0)	194 (47.8)		
Number of Medications					
1–3 pills	101 (49.5)	113 (55.9)	214 (52.7)	1.684	0.194
>3 pills	103 (50.5)	89 (44.1)	192 (47.3)		
Number of Complications					
0	99 (48.5)	124 (61.4)	223 (54.9)	7.024	**0.030**
1	84 (41.2)	60 (29.7)	144 (35.5)		
2 or more	21 (10.3)	18 (8.9)	39 (9.6)		
Exercise					
<3 days per week	143 (70.1)	96 (47.5)	239 (58.9)	21.359	**<0.001**
≥3 days per week	61 (29.9)	106 (52.5)	167 (41.1)		
Alcohol					
<14 units per week	190 (93.1)	185 (91.6)	375 (92.4)	0.347	0.556
≥14 units per week	14 (6.9)	17 (8.4)	31 (7.6)		
Smoking					
Not smoking	197 (96.6)	196 (97.0)	393 (96.8)	0.070	0.792
Smoking	7 (3.4)	6 (3.0)	13 (3.2)		
Intake of Fruit and Vegetable					
<4 servings per day	152 (74.5)	150 (74.3)	302 (74.4)	0.003	0.954
≥4servings per day	52 (25.5)	52 (25.7)	104 (25.6)		
Compliance with low salt diet					
Compliant	137 (67.2)	123 (60.9)	260 (64.0)	1.730	0.188
Non-compliant	67 (32.8)	79 (39.1)	146 (36.0)		
Body Mass Index					
Underweight	5 (2.5)	9 (4.5)	14 (3.4)	9.630	**0.022**
Normal	57 (27.9)	79 (39.0)	136 (33.5)		
Overweight	75 (36.8)	70 (34.7)	145 (35.7)		
Obese	67 (32.8)	44 (21.8)	111 (27.3)		
Blood Pressure					
Controlled	119 (58.3)	111 (55.0)	230 (56.7)	0.473	0.492
Uncontrolled	85 (41.7)	91 (45.0)	176 (43.3)		
Number of Comorbidity					
0	130 (63.7)	111 (55.0)	241 (59.4)	3.842	0.146
1	61 (29.9)	79 (39.1)	140 (34.5)		
2 or more	13 (6.4)	12 (5.9)	25 (6.2)		
Religious and Spiritual activities					
Slightly	27 (13.2)	19 (9.4)	46 (11.3)	5.130	0.077
Moderately	117 (57.4)	103 (51.0)	220 (54.2)		
Completely	60 (29.4)	80 (39.6)	140 (34.5)		
Median Direct Cost of Care *±IQR (₦)*	10,000 ± 17,313	5550 ± 8316	7000 ± 11,800	14,419.50^M^	**<0.001**
Median Indirect Cost of Care *±IQR (₦)*	2000 ± 4790	1670 ± 3180	1670 ± 3970	19,881.00^M^	0.540
Median Total Cost of Care *±IQR (₦)*	13,200 ± 18,400	7750 ± 8600	10,940 ± 11,770	15,062.00^M^	**<0.001**

X^2^ –Chi-square test, *--T-test, ^f^ –Fischer's exact test, ^M^ –Mann-Whitney U test, SD—Standard deviation, IQR- Interquartile Range, P-values < 0.05 are significant (bold).

Table 2 shows that patients from FTH had a significant higher HRQoL physical domain score (STH, 58.98 ± 13.42; FTH, 71.83 ± 18.76) (*p* < 0.001) and overall domains score (STH, 64.85 ± 12.65; FTH, 69.16 ± 18.03) (*p* = 0.006) than those from STH. Other domains of HRQoL score were equally higher among patients in FTH but with no significant difference (*p* > 0.05). Table 3 summarizes the significant factors (*p* < 0.05) in STH and FTH after bivariate analysis. Table 4 shows a significant inverse relationship between cost (direct cost, indirect cost and total cost of care) and all the domains of HRQoL among hypertensive patients in FTH (*P* < 0.005) and also the same relationship between cost (direct cost and total cost of care) and overall and psychological domains among patients in STH (*P* < 0.01).

**Table 2:** Health-related quality of life scores of patients.

Variable	Health Facility		T	p-value
	STH (n = 204)	FTH (n = 202)		
	Mean ± SD	Mean ± SD		
Physical domain	58.98 ± 13.42	71.83 ± 18.76	−7.932	**<0.001**
Psychological domain	67.78 ± 13.18	70.36 ± 15.54	−1.805	0.072
Social domain	66.48 ± 11.98	69.19 ± 17.09	−1.850	0.065
Environmental domain	64.69 ± 11.25	66.58 ± 11.31	−1.686	0.093
Overall domain	64.85 ± 12.65	69.16 ± 18.03	−2.783	**0.006**

SD– Standard deviation, T–T-test, P-values < 0.05 are significant (bold).

**Table 3:** Summary of significant variables after bivariate analysis in all the domains.

Domain Variable	Overall		Physical		Psychological		Social		Environmental	
	STH	FTH	STH	FTH	STH	FTH	STH	FTH	STH	FTH
Age		√	√	√		√		√	√	√
Sex										
Level of Education										
Marital status	√	√		√		√	√			√
Family Type										
Household Size			√	√						
Locality of Residence	√	√	√	√	√	√	√	√	√	√
Living Arrangement		√	√	√	√	√			√	√
Occupation			√							
Income	√					√		√		√
Socioeconomic status										
Length of Diagnosis		√	√	√		√	√			
Number of Medications			√				√			
Number of complications		√	√	√		√		√	√	√
Exercise		√	√	√	√	√	√	√		√
Alcohol										
Smoking	√	√	√	√	√					
Intake of Fruit and Vegetable										
Compliance with low salt diet										
Body Mass Index										
Blood Pressure										
Number of Comorbidities										
Religious and Spiritual activities	√		√		√	√	√		√	

√--significant factor (p < 0.05).

**Table 4:** Relationship between cost of care and HRQoL.

Variable	Health Facility			
	STH (n = 204)		FTH (n = 202)	
Overall Domain				
	**r**	**p-value**	**r**	**p-value**
*Direct Cost of Care*	−0.248	**<0.001**	−0.398	**<0.001**
*Indirect Cost of Care*	−0.046	0.512	−0.209	**0.003**
*Total Cost of Care*	−0.224	**0.001**	−0.378	**<0.001**

Physical Domain				
	**r**	**p-value**	**r**	**p-value**
*Direct Cost of Care*	−0.135	0.054	−0.488	**<0.001**
*Indirect Cost of Care*	−0.067	0.342	−0.360	**<0.001**
***Total Cost of Care***	−0.131	0.063	−0.486	**<0.001**

Psychological Domain				
	**r**	**p-value**	**r**	**p-value**
*Direct Cost of Care*	−0.191	**0.006**	−0.473	**<0.001**
*Indirect Cost of Care*	−0.097	0.166	−0.371	**<0.001**
***Total Cost of Care***	−0.185	**0.008**	−0.475	**<0.001**

Social Domain				
	**r**	**p-value**	**r**	**p-value**
*Direct Cost of Care*	−0.099	0.159	−0.470	**<0.001**
*Indirect Cost of Care*	0.065	0.352	−0.450	**<0.001**
***Total Cost of Care***	−0.072	0.308	−0.489	**<0.001**

Environmental Domain				
	r	p-value	r	p-value
*Direct Cost of Care*	−0.085	0.228	−0.292	**<0.001**
*Indirect Cost of Care*	−0.063	0.374	−0.217	**0.002**
***Total Cost of Care***	−0.086	0.220	−0.291	**<0.001**

*r—*Correlation coefficient, P-values < 0.05 are significant (bold)*.*

Table 5 shows the results of multiple linear regression analysis of the overall and physical domain of HRQoL. It was revealed that for every additional ₦1 rise in the income of STH patient, there is 0.3 increase in their overall domain HRQoL score while holding other variables constant (B = 0.301; 95% CI ≤0.001–3.106; *p* = 0.002). Other predictors of overall domain of HRQoL in STH included being married, residence in an urban area, and complete involvement in spiritual and religious activities. In FTH, overall domain HRQoL score decreased by 0.2 among patients for each additional year in age while holding other variables constant (B = −0.189; 95% CI = −0.349 to −0.029; *p* = 0.021). Locality of residence and number of complications were other predictors of the overall domain of HRQoL in FTH.

**Table 5:** Multiple linear regressions relating HRQoL scores in overall domain and physical domain to predictor variables.

Variables	Health Facility							
	STH (n = 204)				FTH (n = 202)			
	B	p-value	95%CI		B	p-value	95%CI	
			Lower	Higher			Lower	Higher
Overall domain								
Age (years)	–	–	–	–	−0.189	**0.021**	−0.349	−0.029
Marital Status (Married)	4.319	**0.026**	0.513	8.125	1.510	0.568	−3.701	6.721
Locality of Residence (Rural)^REF^								
Semi-urban	2.914	0.300	−2.613	8.442	0.204^**S**^	**0.001**	0.011	2.441
Urban	8.332	**<0.001**	4.187	12.476	5.029	**0.038**	0.289	9.769
Living Arrangement (Not living alone)	–	–	–	–	4.793	0.264	−3.641	13.228
Income (₦)	0.301^**S**^	**0.002**	<0.001	3.106	–	–	–	–
Length of Diagnosis (1–4 years)	–	–	–	–	3.851	0.080	−0.459	8.162
Number of Complications (0)^REF^								
1	–	–	–	–	−0.424^**s**^	**<0.001**	−2.586	−0.010
2 or more	–	–	–	–	−15.235	**0.001**	−24.368	−6.101
Exercise (≥3 days per week)	–	–	–	–	4.011	0.073	−0.371	8.393
Smoking (Yes-Smoking)	−6.500	0.236	−17.236	4.286	0.099^**S**^	0.082	−1.344	22.346
Religious and Spiritual activities (Slightly)^REF^								
Moderately	4.017	0.118	−1.029	9.064	–	–	–	–
Completely	5.498	**0.040**	0.242	10.754	–	–	–	–
Direct COC	0.000	0.245	0.000	0.001	−< 0.001	0.432	- < 0.001	0.000
Indirect COC	–	–	–	–	<0.001	0.654	−0.001	<0.001
Total Cost of Care	−0.001	0.117	−0.001	0.000	–	–	–	–
Physical domain								
Age (years)	−0.144	0.042	−0.284	−0.005	−0.419	**<0.001**	−0.565	−0.272
Marital Status (Married)	–	–	–	–	3.564	0.141	−1.196	8.323
Household Size (1–4 persons)	2.709	0.138	−0.879	6.296	5.427	**0.007**	1.496	9.359
Locality of Residence (Rural)^REF^								
Semi-urban	−0.352	0.900	−5.891	5.186	−3.885	0.083	−8.280	0.510
Urban	2.983	0.148	−1.067	7.032	1.631	0.475	−2.863	6.124
Living Arrangement (Not living alone)	2.793	0.201	−1.499	7.085	−0.563	0.887	−8.373	7.246
Occupation (Retired/Unemployed)^REF^								
Formal	−0.260^S^	**0.004**	−1.195	−0.223	–	–	–	–
Informal	−0.260^**S**^	**0.002**	−1.259	−0.295	–	–	–	–
Length of Diagnosis (1–4 years)	2.450	0.198	−1.289	6.189	−0.164^**s**^	**0.003**	−1.012	−0.021
Number of Medications (1–3 pills)	8.681	**<0.001**	5.252	12.110	–	–	–	–
Number of Complications (0)^REF^								
1	0.183^**S**^	**0.006**	0.146	0.850	−10.082	**<0.001**	−14.816	−5.347
2 or more	−5.262	0.075	−11.068	0.545	−30.661	**<0.001**	−39.578	−21.745
Exercise (≥3 days per week)	7.867	**<0.001**	3.701	12.033	4.643	**0.023**	0.639	8.647
Smoking (Yes-Smoking)	−18.232	**<0.001**	−27.865	−8.600	−13.535	**0.016**	−24.504	−2.566
Religious and Spiritual activities (Slightly)^REF^								
Moderately	−2.417	0.388	−7.923	3.089	–	–	–	–
Completely	1.431	0.631	−4.436	7.298	–	–	–	–
Direct COC	–	–	–	–	–	–	–	–
Indirect COC	–	–	–	–	−0.001	**<0.001**	−0.002	- < 0.001
Total Cost of Care	–	–	–	–	<0.001	0.609	0.000	<0.001

B – Beta Coefficient, 95% CI - 95% Confidence Interval, ^S^ – Standardized Beta Coefficient, COC – Cost of Care, − - Excluded Variable, ^REF^- Reference Category, P-values < 0.05 are significant (bold).

In STH, patients who smoke had 18.2 reduction in their physical domain HRQoL score than those that do not smoke after controlling for other variables (B = −18.232; 95% CI = −27.865 to −8.600; *p* < 0.001). Occupation, number of medications, exercise and having one complication were other predictors of the physical domain of HRQoL in STH. In FTH, physical domain of HRQoL score decreased by 0.4 among patients in FTH for each additional year in age while holding other variables constant (B = −0.419; 95% CI = −0.565 to −0.272; p < 0.001). Other predictors of the physical domain of HRQoL in FTH were household size, length of diagnosis, number of complications, exercise, smoking and indirect cost.

Table 6 shows the results of multiple linear regression analysis of the psychological, social and environmental domains of HRQoL. In STH, patients with complete involvement in spiritual and religious activities had 6.5 increase in their psychological domain HRQoL score than those with slight involvement after controlling for other variables (B = 6.461; 95% CI = 1.814–11.108; *p* = 0.007). Residence in urban area, living arrangement, exercise and smoking were other predictors of the psychological domain of HRQoL in STH. In FTH, patients with 1 complication and 2 or more complications had 14.9 and 15.9 reduction respectively in their psychological domain HRQoL score than those without complication after controlling for other variables (one complication: B = −14.886; 95% CI = −18.711 to −11.061; *p* < 0.001, 2/more complications: B = −15.874; 95% CI = −23.280 to −8.468; p < 0.001). Other predictors of the psychological domain in FTH were exercise, residing in an urban area, and indirect cost.

**Table 6:** Multiple linear regressions relating HRQoL scores in psychological domain, social domain and environmental domain to predictor variables.

Variable	Health Facility							
	STH (n = 204)				FTH (n = 202)			
	B	p-value	95%CI		B	p-value	95%CI	
			Lower	Higher			Lower	Higher
Psychological domain								
Age (years)	–	–	–	–	−0.063	0.320	−0.187	0.061
Marital Status (Married)	–	–	–	–	1.535	0.476	−2.705	5.776
Locality of Residence (Rural)^REF^								
Semi-urban	0.872	0.723	−3.981	5.725	−0.088	0.969	−4.546	4.371
Urban	14.073	**<0.001**	10.435	17.710	5.980	**0.001**	2.362	9.598
Living Arrangement (Not living alone)	7.424	**<0.001**	3.949	10.899	−2.140	0.519	−8.665	4.386
Income (₦)	–	–	–	–	0.000	0.110	0.000	0.000
Length of Diagnosis (1–4 years)	–	–	–	–	2.565	0.149	−0.930	6.061
Number of Complications (0)^REF^								
1	–	–	–	–	−14.886	**<0.001**	−18.711	−11.061
2 or more	–	–	–	–	−15.874	**<0.001**	−23.280	−8.468
Exercise (≥3 days per week)	5.553	**0.001**	2.340	8.766	−0.149^**s**^	**0.046**	−9.135	−0.093
Smoking (Yes-Smoking)	−12.748	**0.004**	−21.256	−4.240	–	–	–	–
Religious and Spiritual activities (Slightly)^REF^								
Moderately	−0.301	0.897	−4.870	4.269	−2.862	0.434	−10.056	4.332
Completely	6.461	**0.007**	1.814	11.108	8.381	0.051	−0.018	16.780
Direct COC	0.000	0.120	0.000	0.001	- < 0.001	0.203	- < 0.001	0.000
Indirect COC	–	–	–	–	−0.001	**<0.001**	−0.001	- < 0.001
Total Cost of Care	0.000	0.065	−0.001	0.000	–	–	–	–
Social domain								
Age (years)	–	–	–	–	−0.152	0.077	−0.320	0.017
Marital Status (Married)	2.692	0.117	−0.684	6.069	–	–	–	–
Locality of Residence (Rural)^REF^								
Semi-urban	3.118	0.229	−1.981	8.217	1.713	0.514	−3.448	6.874
Urban	9.754	**<0.001**	6.064	13.443	2.273	0.371	−2.729	7.275
Income (₦)	–	–	–	–	0.195^**s**^	**0.003**	<0.001	0.304
Length of Diagnosis (1–4 years)	3.276	0.051	−0.019	6.570	–	–	–	–
Number of Medications (1–3 pills)	2.432	0.122	−0.659	5.522	–	–	–	–
Number of Complications (0)^REF^								
1	–	–	–	–	1.471	0.555	−3.437	6.380
2 or more	–	–	–	–	−3.606	0.427	−12.540	5.328
Exercise (≥3 days per week)	0.174	0.923	−3.362	3.709	−0.059	0.980	−4.739	4.621
Religious and Spiritual activities (Slightly)^REF^								
Moderately	13.884	**<0.001**	9.322	18.446	–	–	–	–
Completely	14.630	**<0.001**	9.734	19.527	–	–	–	–
Direct COC	–	–	–	–	0.000	0.113	0.000	0.000
Indirect COC	–	–	–	–	−0.001	**0.001**	−0.001	- < 0.001
Total Cost of Care	–	–	–	–	–	–	–	–
Environmental domain								
Age (years)	0.200	**<0.001**	0.094	0.305	−0.071	0.202	−0.182	0.039
Marital Status (Married)	–	–	–	–	−0.741	0.697	−4.495	3.013
Locality of Residence (Rural)^REF^								
Semi-urban	4.868	**0.038**	0.275	9.461	3.257	0.063	−0.182	6.697
Urban	8.841	**<0.001**	5.513	12.170	8.596	**<0.001**	5.228	11.963
Living Arrangement (Not living alone)	3.684	**0.035**	0.262	7.106	1.128	0.716	−4.969	7.226
Income (₦)	–	–	–	–	0.243^**s**^	**<0.001**	<0.001	0.360
Number of Complications (0)^REF^								
1	0.554	0.703	−2.302	3.409	0.245	0.889	−3.214	3.704
2 or more	−11.187	**<0.001**	−15.765	−6.609	−8.125	**0.014**	−14.583	−1.668
Exercise (≥3 days per week)	–	–	–	–	5.292	**0.001**	2.173	8.411
Religious and Spiritual activities (Slightly)^REF^								
Moderately	−0.122	0.952	−4.110	3.865	–	–	–	–
Completely	6.879	**0.002**	2.616	11.142	–	–	–	–
Direct COC	–	–	–	–	0.000	0.490	0.000	0.000
Indirect COC	–	–	–	–	0.000	0.098	−0.001	0.000
Total Cost of Care	–	–	–	–	–	–	–	–

B – Beta Coefficient, 95% CI - 95% Confidence Interval, ^S^ – Standardized Beta Coefficient, COC – Cost of Care, − - Excluded Variable, ^REF^- Reference Category, P-values < 0.05 are significant (bold).

It was also found that in STH, being resident in an urban area as well as involvement in religious and spiritual activities were predictors of the social domain of HRQoL while income and indirect cost were the predictors of social domain of HRQoL in FTH. There was a decrease of 0.001 in the social domain HRQoL score for every ₦1 increase in the indirect cost while holding other variables constant (B = −0.001; 95% CI = −0.001 – −< 0.001; *p* = 0.001).

Lastly, STH patients that were not living alone had 3.7 increase in their environmental domain HRQoL score than those living alone after controlling for other variables (B = 3.684; 95%CI = 0.262–7.106; *p* = 0.035). Age, locality of residence, having 2 or more complications, and complete involvement in spiritual and religious activities were other predictors of environmental domain in STH. In FTH, the predictors of the environmental domain were being resident in an urban area, income, exercise, and having 2 or more complications. Patients with 2 or more complications had a reduction of 8.1 in their environmental domain HRQoL score than those without complication after controlling for other variables (B = −8.125; 95% CI = −14.583 to −1.668; *p* = 0.014).

## 4 Discussion

This study assessed and compared HRQoL and its predictors among hypertensive patients accessing care in a STH and a FTH in Nigeria. It was found that FTH hypertensive patients had better HRQoL than the STH patients. Identified predictors of HRQoL were age, locality of residence, income, number of complications, exercise and smoking in both hospitals. Other predictors were marital status, living arrangement, occupation, number of medications, and involvement in religious and spiritual activities among patients in STH; household size, length of diagnosis, and indirect cost among patients in the FTH. Lastly, there was an inverse relationship between cost and HRQoL of hypertension.

The FTH had a higher proportion of hypertensive patients without complications and with normal BMI. This finding may be a result of more personnel, better infrastructure and equipment, as well as other resources allocated to the hospital as these influence the quality of care received by the patients. Also, the median cost of care is lower in FTH than in STH which may improve access to care in FTH, thereby leading to a more favorable clinical outcome for the FTH patients.

Most authors assessed HRQoL of hypertensive patients in comparison with normotensive population [4]. However, in this study, HRQoL was compared among two groups of hypertensive patients attending two different hospitals. It was observed that HRQoL was better among patients in FTH than in STH. This may not be surprising given that FTH had a higher proportion of hypertensive patients who were without complications and with normal BMI. Absence of comorbidity, complications as well as normal BMI among hypertensive patients have been found to have a significant positive effect on the HRQoL of hypertensive patients [5,9,10,13]. This finding may also be linked with the disparity in the resource allocation and health budget provisions of the hospitals which may affect both the quantity and quality of healthcare service delivery in the institutions. The mean HRQoL scores in this study are comparable with that of a study carried out in Oyo State, Nigeria [5]. Comparison with studies outside Nigeria may be difficult because scales other than WHOQoL-BREF were used by most of these studies.

Furthermore, this study identified the predictors of HRQoL among hypertensive patients. Age was identified as a predictor of HRQoL among hypertensive patients in both hospitals. Overall and physical domain HRQoL scores decreased by 0.2 and 0.4 among patients in FTH for each additional year in age respectively. Previous studies have documented a higher HRQoL score among younger hypertensive patients [4,8,10,12,13]. During the aging process, health hazards may arise as a result of psychological and physiological changes, making the individual more vulnerable to chronic diseases including hypertension which can affect HRQoL [4]. However, Baune *et al* and Saleem *et al* in their studies did not find a significant association between age and HRQoL among hypertensive patients in Asia [3,11]. This may be due to the racial and other differences in the populations that were studied.

This study found locality of residence as a predictor of HRQoL in both groups. Being resident in an urban area is a positive predictor of higher HRQoL in all domains except for physical in STH in addition to social and physical in FTH. This is consistent with reports from other studies [3,8,13] but contrary to the findings by Zhou *et al* [32]. Additionally, smaller household size predicted a higher physical domain HRQoL score in FTH, unemployment predicted a lower physical domain HRQoL score in STH while income was a positive predictor of HRQoL in both hospitals. Previous studies have shown that unemployment worsens HRQoL [3,[8], [9], [10],^13^]. Similarly, lower income and socioeconomic status had a negative effect on HRQoL [4,5,[8], [9], [10], [11], [12],^16^]. However, this study did not identify socioeconomic status as a factor that affects HRQoL of hypertensive patients.

For the finding about living in urban area, disparities may be enhanced by more infrastructure and social amenities in these areas. In Nigeria, the average household size is slightly higher in rural than in urban areas [33]. Unemployment rate is also higher in rural than urban areas. Analysis of youth unemployment by geographical location indicated that unemployment was mostly in rural areas and is rapidly growing [34]. A high unemployment rate will definitely reduce household income. Competing demand for this small household revenue, especially in the face of a large household size could lead to household poverty. This vicious cycle of unemployment and poverty could limit access to health care and subsequently worsen hypertension in patients thereby reducing the quality of life. The economic vulnerability also impacts health and HRQoL in terms of affordability of resources such as food, good housing, transportation, and other daily needs [12]. This finding suggested that increasing employment rate in rural areas and the nation at large will contribute to the improvement of the HRQoL of hypertensive patients.

As regards the clinical variables, shorter length of diagnosis and absence or reduced number of complications were predictive of higher HRQoL among patients in STH and FTH. These results are consistent with findings from previous studies where shorter duration of disease [4,10] and absence of complications [5,9] have a significant positive effect on HRQoL of hypertensive patients. This study showed the negative impact of an increased number of medications on HRQoL. This finding supported studies done by Adedapo *et al* and Al-jabi *et al* [5,10]. However, it contradicted a study by Ghimire *et al* that reported a better HRQoL with increasing number of antihypertensive medications [12]. Another study from Brazil reported no association between number of medications and HRQoL [4]. Pill burden, resulting from antihypertensive combinations, makes the daily routine of medication taking complex and can be a barrier to optimal adherence [35], which is associated with poor HRQoL [36]. Another reason for the negative effect of an increased number of medications on HRQoL may result from the side effects of drugs. Drugs offered to patients should have minimal side effects with maximal benefits.

Adequate exercise was a positive predictor of physical and psychological domains in both hospitals. This finding is consistent with that of Zhang *et al* [13]. Smoking is another modifiable risk factor, that increases the risk of cardiovascular morbidity and may lead to further deterioration in HRQoL of hypertensive patients [29]. In this study, smoking is a predictor of lower HRQoL in both STH and FTH. This result is similar to that of a study in China [13]. The study also found a negative association between HRQoL and alcohol consumption. The finding on alcohol use is however not consistent with the finding from the present study, which did not find any association between alcohol use and HRQoL. The difference in findings may result from the level of alcohol use that was assessed by the studies. The previous study assessed if patients had ever used alcohol or otherwise while the present study assessed if patients used alcohol safely or dangerously. The disparity may also result from the difference in alcohol consumption between the two populations that were studied.

This study identified complete involvement in religious and spiritual activities as a predictor of higher HRQoL in all except the physical domain among hypertensive patients in STH. This result is similar to the finding of a systematic review [18]. It is nonetheless different from the findings by Westlake *et al* [37]. Religious and spiritual practices influence coping mechanisms in dealing with various chronic illnesses. In many patients with chronic diseases, religious and spiritual activities are important and highly personal aspects of their disease experience and coping strategy. Religiousness has been shown to enhance self-esteem, generate positive emotions, and promote positive self-care practices by encouraging individuals to refrain from unhealthy lifestyle practices, which in turn fosters well-being and HRQoL [18].

Another key finding in this study is the establishment of an inverse relationship between cost and HRQoL in both STH and FTH. Studies done among other chronic disease patients also found an inverse association between cost and HRQoL [[19], [20], [21]]. Hypertensive patients with poor HRQoL were more likely to be older with longer duration of disease. They were also more likely to have one or more complications and increased need for medical interventions including drugs. Increasing age, presence and increasing number of complications, longer duration of disease and increased need for medical interventions have been previously documented as determinants of higher healthcare cost [38,39] and could be the reason for the negative association between cost and HRQoL observed in this study.

Lastly, this study found that poor HRQoL in patients with hypertension is associated with higher healthcare expenditure. The study also revealed that poor HRQoL is associated with unemployment. An unemployed individual due to financial hardship is likely to experience difficulty in accessing health care especially in the setting of increased healthcare cost, thereby resulting in deterioration of health and well-being. This deterioration in health and well-being could further negatively impact the HRQoL of the individual leading to a negative vicious cycle. Unfortunately, formal health insurance which could help minimize the economic burden of this disease is lacking for many Nigerians [40,41], making them bear the full brunt of these economic impacts. These huge economic effects have significant implications for healthcare policy in the country.

An important limitation of this study is its cross-sectional design. Cross-sectional design could limit the extent to which inferences can be drawn with respect to causal relationships among variables. Also, this study depends on asking subjects about their past experiences, which makes it prone to recall bias; however, this was mitigated by asking questions in the last one month preceding the study.

## 5 Conclusion

HRQoL was better among hypertensive patients in FTH than STH. Socio-demographic, clinical and other variables associated with HRQoL in both hospitals were identified. Additionally, an inverse relationship was found between cost (direct, indirect and total cost of care) and HRQoL of hypertension. There is a need for government to support hypertensive patients in STH in order to reduce the inequality of low HRQoL among them. Identified predictors should be modified in such a way (e.g cessation of smoking, adequate exercise, involvement in religious and spiritual activities, low pill burden) that would improve the HRQoL of these patients.

## Funding

No funds, grants, or other support was received for this study.

## Authors' contribution statements

Tope Michael Ipinnimo conceptualized and designs the research. Literature search and data collection was carried out by Tope Michael Ipinnimo, Bolade Folasade Dele-Ojo, Olarinde Jeffrey Oluwademilade, Taofeek Adedayo Sanni, Olumide Temitope Asake, Blessing Waibi Daramola, Adetunji Olamide Fadipe. Statistical analysis was conducted by Tope Michael Ipinnimo while results were interpreted by Tope Michael Ipinnimo, Kayode Rasaq Adewoye, Kabir Adekunle Durowade, Olusegun Elijah Elegbede, John Olujide Ojo. The manuscript was drafted and critically revised for important intellectual content by Tope Michael Ipinnimo, Kayode Rasaq Adewoye, Kabir Adekunle Durowade and Oladele Ademola Atoyebi. Editing and critical review was by all authors. The final draft was read and approved by all authors.

## Ethical approval and consent to participate

Ethical approval for the study was sought and obtained from the Ethics and Research Review Committee of Federal Teaching Hospital, Ido-Ekiti, Nigeria. Written informed consent was obtained from all the research subjects.

## Availability of data and material

The datasets used and/or analyzed during the current study are available from the corresponding author on request.

## Declaration of Competing Interest

The authors have no competing interests to declare that are relevant to the content of this article.
